# Effects of *Lonicera japonica* Extract with Different Contents of Chlorogenic Acid on Lactation Performance, Serum Parameters, and Rumen Fermentation in Heat-Stressed Holstein High-Yielding Dairy Cows

**DOI:** 10.3390/ani14081252

**Published:** 2024-04-22

**Authors:** Fengtao Ma, Junhao Liu, Shengli Li, Peng Sun

**Affiliations:** 1 State Key Laboratory of Animal Nutrition and Feeding, Institute of Animal Science, Chinese Academy of Agricultural Sciences, Beijing 100193, China; fengtaoma@163.com (F.M.);; 2 State Key Laboratory of Animal Nutrition and Feeding, Beijing Engineering Technology Research Center of Raw Milk Quality and Safety Control, College of Animal Science and Technology, China Agricultural University, Beijing 100193, China

**Keywords:** *Lonicera japonica* extract, chlorogenic acid, heat stress, antioxidant status, immune function, rumen fermentation parameters, dairy cow

## Abstract

**Simple Summary:**

The susceptibility of high-yielding dairy cows to heat stress results in significant economic losses for the dairy farming industry. In this study, we evaluated the effects of *Lonicera japonica* extract (LJE) with different chlorogenic acid (CGA) contents on lactation performance, serum biochemical and antioxidant indicators, immune factors, and rumen fermentation parameters in heat-stressed high-yielding dairy cows. After feeding dairy cows for 56 days, we found that the addition of LJE promoted lactation performance and rumen fermentation in high-yielding cows experiencing heat stress. It also increased the levels of IgG and the activities of glutathione peroxidase, superoxide dismutase, and total antioxidant capacity, while reducing the concentrations of creatinine, malondialdehyde and inflammatory factors in the serum. Furthermore, while maintaining a consistent level of CGA content, the effects of addition from both types of LJE are similar. These findings demonstrate that LJE exhibits remarkable efficacy in alleviating heat stress and enhancing the lactation performance of dairy cows during hot summers, with CGA serving as the effective ingredient responsible for its anti-heat stress properties.

**Abstract:**

This examined the effects of *Lonicera japonica* extract (LJE) with different chlorogenic acid (CGA) contents on lactation performance, antioxidant status and immune function and rumen fermentation in heat-stressed high-yielding dairy cows. In total, 45 healthy Chinese Holstein high-yielding dairy cows, all with similar milk yield, parity, and days in milk were randomly allocated to 3 groups: (1) the control group (CON) without LJE; (2) the LJE-10% CGA group, receiving 35 g/(d·head) of LJE-10% CGA, and (3) the LJE-20% CGA group, receiving 17.5 g/(d·head) of LJE-20% CGA. The results showed that the addition of LJE significantly reduced RT, and enhanced DMI, milk yield, milk composition, and improved rumen fermentation in high-yielding dairy cows experiencing heat stress. Through the analysis of the serum biochemical, antioxidant, and immune indicators, we observed a reduction in CREA levels and increased antioxidant and immune function. In this study, while maintaining consistent CGA content, the effects of addition from both types of LJE are similar. In conclusion, the addition of LJE at a level of 4.1 g CGA/(d·head) effectively relieved heat stress and improved the lactation performance of dairy cows, with CGA serving as the effective ingredient responsible for its anti-heat stress properties.

## 1. Introduction

The susceptibility of high-yielding dairy cows to heat stress (HS) results in significant economic losses for the dairy farming industry [1,2]. Heat stress reduces milk yield and quality [3], impairs immune function [4], induces oxidative stress and systemic inflammation [5,6], and disrupts metabolism and energy balance in dairy cows [7,8]. The rumen is a sensitive micro-ecosystem easily influenced by external factors. Heat stress affects feeding behavior and feed intake, alters rumen fermentation [9], and impairs rumen function [7]. Despite the use of evaporative cooling systems like fans and soakers to dissipate heat [10], heat stress remains a major challenge for the dairy industry. Therefore, it is imperative to research and develop natural, green, and safe nutritional additives as an effective strategy for alleviating heat stress to ensure high level of milk production in dairy cows during hot summers.

Chinese herbal additives have nutritional and medical values and few toxic side effects; as such, they have been widely used to improve livestock production during high ambient temperatures in hot summers [11,12]. *Lonicera japonica* extract (LJE) is abundant in chlorogenic acid (CGA) and exhibits a spectrum of pharmacological activities, encompassing anti-inflammatory, immunomodulatory, and antioxidant effects. Its versatility extends to its application as both a pharmaceutical agent and a constituent in health-promoting food products [13]. Therefore, LJE has the potential to relieve heat stress in dairy cows [14,15]. Our previous research found that different doses of LJE-10% CGA (14 g/(d·head), 28 g/(d·head), and 56 g/(d·head)) improved the antioxidant capacity and immune function of heat-stressed dairy cows, but had no significant effect on their lactation performance [16]. Based on that, the optimal dose of LJE-10% CGA to be added was determined to be 35 g/(d·head) through quadratic curve fitting between key indicators and the dosage, calculating the corresponding optimal dose for each indicator and averaging the values. Many studies have speculated that CGA may be the main effective ingredient for the dual nutritional and medicinal effects of LJE. Palíková et al. [17] found that the CGA, isolated from Lonicera japonica, alleviated oxidative damage in rat liver microsome and human umbilical vein endothelial cells. Zhao et al. [18] found a potential relationship between CGA and the reduction of inflammatory responses in perinatal cows through LJE. Available substantial evidence supports that CGA has regulatory effects on key pathways involved in oxidative stress and inflammatory responses [19,20]. However, it remains unclear whether CGA is the effective ingredient in LJE for alleviating heat stress in dairy cows. This study used two types of LJE, with different concentrations of CGA (10% and 20% CGA in LJE), to compare their effects on lactation performance, serum biochemical and antioxidant indicators, immune factors, and rumen fermentation parameters in heat-stressed high-yielding dairy cows while providing consistent CGA content. By validating the effectiveness of the addition of LJE at the optimal dose, this research aimed to provide data supporting CGA from LJE as the effective ingredient in alleviating heat stress in dairy cows.

## 2. Materials and Methods

This study was conducted from 15 June 2022 to 2 August 2022 at Hebei Junyuan Dairy Farm (Shijiazhuang, Hebei Province, China). All procedures were approved by the Institute of Animal Science, Chinese Academy of Agricultural Sciences (Beijing, China). All animals in this study were raised according to the standards established by the Institute of Animal Science, Chinese Academy of Agricultural Sciences (Beijing, China).

### 2.1. Animals, Diets, and Experimental Design

In total, 45 multiparous high-yielding Holstein dairy cows, characterized by their parity (2.3 ± 0.3), average daily milk yield (35.0 ± 1.0 kg/d), and days in milk (95 ± 5 d), were randomly allocated to 3 groups (n = 15). The groups were as follows: (1) the control group (CON) without LJE; (2) the LJE-10% CGA group, receiving 35 g/(d·head) of LJE-10% CGA (LJE with 11.75% chlorogenic acid, containing 4.1 g CGA/(d·head)), and (3) the LJE-20% CGA group, receiving 17.5 g/(d·head) of LJE-20% CGA (LJE with 23.62% chlorogenic acid, containing 4.1 g CGA/(d·head)). LJE-10% CGA and LJE-20% CGA, provided by Shanxi Zhongxin Bio-Tech Co. Ltd. (Xi’an, Shanxi Province, China), were identified according to the standard of the Pharmacopeia of the People’s Republic of China. The dairy cows were individually fed with the same TMR (Table 1), formulated to meet the nutrient requirements by NRC (2001). This TMR was administered to cows 3 times daily after milking sessions at 0630, 1330, and 2030 h, with a refusal rate of 5 to 10%. Refusals were regularly removed and quantified each morning. LJE was applied as a top-dressing on the TMR during the morning feeding. The experiment spanned 10 wk, preceded by a 2 wk pre-feeding period. Housing was provided in a mechanically ventilated tiestall barn, with cows divided into 3 groups of 15, and they had continuous access to water throughout the study. Prior to the trial, the dairy cows experienced heat stress, with the THI averaging 78.5 and the ambient temperature averaging 25.7 °C for 2 weeks. In the week leading up to the experiment, the rectal temperature (RT) and respiration rate (RR) averaged 39.0 °C and 84 breaths/min, respectively.

### 2.2. Sampling and Analysis

During the experimental period, the temperature (T) and relative humidity (RH) within the barn were recorded 3 times daily (at 6:00, 14:00, and 22:00) by suspending a digital thermohygrometer (Beijing Yaguang Equipment Co. Ltd., Beijing, China) 1.5 m above the ground. THI was computed using the equation established by Dikmen and Hansen (2009): THI = (1.8 × T + 32) − [(0.55 − 0.0055 × RH) × (1.8 × T − 26)], where T represents the ambient temperature (°C) and RH denotes the relative humidity (%). The RT of cows was monitored daily at 7:30, 14:30, and 21:30 using glass mercury thermometers (Nasco, Ft. Atkinson, WI, USA), and the RR was also measured 3 times per day at the same intervals by counting flank movements for 1 min.

The daily dry matter intake (DMI) for individual cows was determined by subtracting feed refusals from the offered TMR on a DM basis. TMR samples were collected 3 days per week during the treatment period to assess DM content. These samples were combined, dried for 48 h at 65 °C, and ground to 1 mm using a fodder grinder. The DM content was then determined by drying in an oven for 4 h at 105 °C until a constant weight was achieved (AOAC International, 2005; method 930.15) [22]. Crude protein and ether extract were analyzed following the AOAC International methods 976.05 and 4.5.05 [23], respectively. The neutral detergent fiber and acid detergent fiber contents were determined according to Van Soest et al. [24]. Calcium and phosphorus levels were measured using AOAC International methods 985.35 and 986.24 [25], respectively. The composition and nutrient levels of the TMR are detailed in Table 1.

Cows were milked 3 times daily at 5:30, 12:30, and 19:30, with individual milk yield recorded at each milking. Milk samples from each cow were collected over three consecutive milkings and combined weekly at a ratio of 4:3:3 [26]. Bronopol tablets (D&F Control System, San Ranmon Inc., Dublin, ON, Canada) were added to the milk samples as a preservative and then stored at 4 °C. Infrared analysis (Foss MilkoScan 2000, Foss Food Technology Corp., Eden Prairie, MN, USA) was employed to determine milk components, including fat, protein, lactose, total solids (TS), and solids-not-fat (SNF).

Blood samples of 45 dairy cows were collected from the tail root vein before morning feeding (at 6:00 on d 57). After resting at room temperature for 30 min, serum samples were prepared from 3000× *g* at 4 °C for 15 min, and stored in the refrigerator at −20 °C for future analysis:

- Biochemical index: serum creatinine (CREA), uric acid (UA), urea nitrogen (UREA), and total protein (TP) concentrations were measured using an automated biochemical analyzer (Hitachi 7080; Hitachi Valve Ltd., Tokyo, Japan).
- The serum levels of total antioxidant capacity (T-AOC, catalog number A015-1, Colorimetric method), superoxide dismutase (SOD, catalog number A001-3, WST-1 method), glutathione peroxidase (GSH-Px, catalog number A005-1, Colorimetric method), and malondialdehyde (MDA, catalog number A003-1, TBA method) were measured using commercial assay kits (Nanjing Jian Cheng Bioengineering Institute, Nanjing, China) following the manufacturer’s instructions.
- Immunoglobulins and inflammatory cytokines: Bovine serum immunoglobulin (IgA (catalog number CSB-E12018B), IgG (catalog number CSB-E12015B), and IgM (catalog number CSB-E12017B)) and inflammatory cytokine (interleukin (IL-) 6 (catalog number CSB-E12899B), IL-1β (catalog number CSB-E12019B), IL-18 (catalog number CSB-EL011608BO) and tumor necrosis factor (TNF)-α (catalog number CSB-E12020B) concentrations were tested using enzyme-linked immunosorbent assay (ELISA) kits (CUSABIO., Wuhan, China) according to the manufacturer’s instructions.

After 2 h of morning feeding at the end of the experiment (1000 h on d 57), 6 cows from each group were randomly selected for collecting rumen fluid samples using the Stomach tube type ruminal fluid sampler (Wuhan Kolibo Animal Science and Technology Co., Ltd., Wuhan, China) (n = 6). When collecting ruminal fluid from the rumen, the first 200 mL obtained was discarded. The pH value of the ruminal fluid was measured immediately using a portable pH meter (Seven GoTM portable pH meter, Mettler Toledo, Switzerland). Thereafter, the ruminal fluid samples were filtered through four layers of cheesecloth, and the filtered samples were individually collected into a frozen storage tube and preserved at −80 °C for VFA analysis. The VFA (total volatile acid, acetate, propionate, isobutyrate, butyrate, isovalerate and valerate) concentrations in the cultures were analyzed using a gas chromatograph (7890A, Agilent Technologies, Santa Clara, CA, USA). The microbial protein (MCP) yield was determined by measuring urinary purine derivative excretion using a previously reported method [27].

### 2.3. Statistical Analysis

All data were analyzed using SAS version 9.4 (SAS Institute Inc., Cary, NC, USA). Milk yield, milk composition, and DMI were analyzed by unstructured covariance with repeated measurements using the mixed procedure. The measurements of DMI, milk yield, and composition during the pre-treatment period served as covariates for the corresponding treatment period. The statistical model contained fixed effects of the study week, treatment group, the interaction of the week and treatment, and the random effect of cow identity. Serum biochemical indexes, antioxidant indicators, immune indices, and rumen fermentation parameters were analyzed using ANOVA in the GLM procedure of SAS. Physiological parameters were firstly calculated using the mean of each parameter every day and then statistical analysis was conducted using the same procedure as the other variables. Results are presented as least squares means and the standard errors of the mean. Differences of *p* < 0.05 were considered statistically significant, and 0.05 ≤ *p* < 0.1 was referenced as a tendency.

## 3. Results

### 3.1. Measurement of THI and Heat Stress

**Figure 1:**
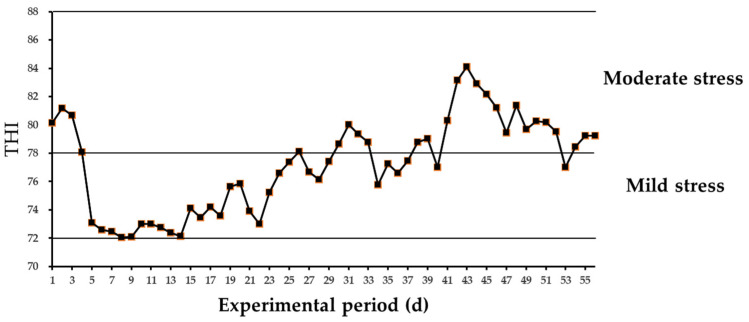
Average temperature–humidity index (THI) pattern in the barn during the experimental period.

As shown in Figure 1, the average THI in the barn during the experimental period was >72. There were 25 days with an average THI exceeding 78, indicating a heat-stressed environment for the dairy cows. As seen in Table 2, the RT of the dairy cows was significantly reduced when supplemented with LJE (*p* < 0.05). However, the RR was not affected by the treatments (*p* > 0.05).

### 3.2. Lactation Performance

The results of the addition of LJE on the DMI and lactation performance of heat-stressed dairy cows are presented in Table 3. The addition of LJE-10% CGA and LJE-20% CGA significantly increased DMI, milk yield, milk protein and lactose contents (*p* < 0.05), and tended to increase milk fat (*p* = 0.09) compared to the CON group.

### 3.3. Serum Biochemical Indexes

As shown in Table 4, the addition of LJE did not affect the concentrations of uric acid, total protein, and urea nitrogen in the serum of dairy cows under heat stress (*p* > 0.05). However, the addition of LJE significantly reduced serum creatinine concentrations compared to the CON group (*p* < 0.05).

### 3.4. Antioxidant Indicators

As shown in Table 5, the addition of LJE-10% CGA and LJE-20% CGA in heat-stressed dairy cows increased serum T-AOC, SOD, and GSH-Px activities compared to the CON group (*p* < 0.05), while reducing serum MDA concentrations (*p* < 0.05).

### 3.5. Serum Immunoglobulins and Inflammatory Cytokines

As shown in Table 6, compared to the CON group, both LJE-10% CGA and LJE-20% CGA groups had higher serum concentrations of IgG content (*p* < 0.05), whereas LJE-10% CGA and LJE-20% CGA reduced the serum concentrations of IL-6, TNF-α, IL-18, and IL-1β compared to the CON group (*p* < 0.05).

### 3.6. Rumen Fermentation Parameters

As shown in Table 7, the addition of LJE-10% CGA and LJE-20% CGA significantly increased rumen pH, MCP, total VFA, and rumen acetate content in heat-stressed cows compared to the CON group (*p* < 0.05).

## 4. Discussion

Dairy cows are susceptible to high summer temperatures. Heat stress begins when the THI exceeds 68 and mild heat stress occurs when the THI exceeds 72 [28]. Our observations during the study period revealed that temperatures consistently exceeded 25 °C and the average THI remained above 72. The CON group of cows exhibited a RT of 39.14 °C and RR of 85.31 breaths/min, indicating that the cows were under heat stress conditions.

During the summer season, dairy cows may experience hyperthermia due to environmental heat stress. RT and RR serve as crucial physiological indicators for assessing heat stress in dairy cows [29]. Dairy cows are considered to be under heat stress when the RR exceeds 60 breaths/min and the RT surpasses 38.5 °C [10]. Even a slight elevation in RT can significantly impact tissue and endocrine functions, thereby severely affecting dairy cow productivity and health [30]. In this study, the addition of LJE-10% CGA and LJE-20% CGA reduced the RT in heat-stressed dairy cows, suggesting that the addition of LJE at a level of 4.1 g CGA/(d·head) for heat-stressed dairy cows can alleviate heat stress by improving physiological conditions.

In heat-stressed dairy cows, high temperatures increase body temperature, prolonging feed retention in the rumen and activating stomach stretch sensors on the rumen walls [31]. This condition affects the anorexia center in the hypothalamus, leading to a reduced appetite and feed intake [32]. Consequently, heat-stressed dairy cows have a lower DMI, resulting in decreased energy intake. Simultaneously, the high temperature environment causes water loss and increased energy metabolism, further elevating energy consumption. As a result, heat-stressed dairy cows experience an imbalance between energy intake and expenditure, ultimately leading to a negative energy balance which impacts lactation performance and overall health [32]. In this study, we found that adding LJE-10% CGA and LJE-20% CGA increased the DMI, milk yield, and milk composition in heat-stressed dairy cows, and there was no significant difference between the two groups. These results indicate that LJE alleviates heat stress by improving the negative energy balance of heat-stressed cows. Furthermore, lactose plays a pivotal role in regulating milk osmotic pressure and is a crucial determinant of milk production. Heat stress-induced insulin resistance in dairy cows, resulting from negative energy balance, can impede lactose synthesis and subsequently leads to reduced milk yield [33]. It is noteworthy that this study observed a significant increase in lactose content, which may be attributed to the effective alleviation of insulin resistance by CGA [34]. Additionally, our previous studies showed that the addition of 14, 28, or 56 g/(d·head) of LJE-10% CGA did not affect the lactation performance, but only showed numerical increases in heat-stressed dairy cows. This study suggested that the addition of LJE containing 4.1 g CGA/(d·head) improved the lactation performance in dairy cows, indicating that CGA is an effective ingredient of LJE in restoring the lactation performance of heat-stressed dairy cows.

Serum CREA is a crucial indicator for assessing amino acid balance and protein metabolism in animals. It accurately reflects significant renal injury, suggesting impaired renal function and indicating that inadequate excretion may lead to renal failure [35,36]. Cowley et al. [37] observed elevated plasma concentrations of CREA in dairy cows experiencing heat stress, possibly due to the negative energy balance caused by heat stress. Tzeng et al. [38] demonstrated that *Lonicera japonica*’s ethanolic extract significantly reduced serum CREA concentration in diabetic rats, indicating an association with CGA in LJE. Meanwhile, Ye et al. [39,40] found that CGA had a protective effect on both diabetic rats and LPS-induced AKI mice by reducing serum CREA concentration and attenuating renal damage. Consistently, our results showed a significant decrease in serum CREA concentration after the addition of LJE with 10% and 20% CGA.

Heat stress in cows elevates their body temperature, increasing metabolic enzyme activity and raising the metabolic rate. This intensifies the response of the sympathetic-adrenal system, leading to the secretion of a large amount of catecholamines by the adrenal glands and generating free radicals that disrupt the balance between oxidative and antioxidative systems. Lipid peroxidation occurs, causing damage to DNA and proteins, ultimately resulting in oxidative stress in cows [41]. Pal et al. [42] found that heat stress leads to a decrease in the activity of T-AOC, GSH-Px, and SOD in the serum of cows, accompanied by a significant increase in MDA content. T-AOC serves as a crucial indicator for assessing the body’s antioxidant capacity, while GSH-Px acts as a key antioxidant enzyme in preventing ROS accumulation. SOD catalyzes the dismutation of superoxide anion radicals, producing oxygen and hydrogen peroxide, representing a vital marker of antioxidant status. MDA levels reflect the extent of lipid peroxidation within tissue cells [43,44,45,46]. In this study, supplementing with two kinds of LJE significantly increased the activity of T-AOC, GSH-Px, and SOD while reducing MDA levels. Similar results were obtained by Zhao et al. [17], who suggested that removal of excess free radicals was attributable to the potent antioxidant effects of CGA contained in LJE, as described in numerous previous research studies [47,48]. In this study, the addition of LJE containing 4.1 g CGA/(d·head) enhanced the antioxidant capacity of heat-stressed cows.

It is well known that heat stress is closely related to the inflammatory response, which increases the production of inflammatory factors (TNF-α, IL-6, IL-1β, and IL-18) [49,50]. The elevation of TNF-α, IL-6, IL-1β, and IL-18 can exacerbate the systemic inflammatory response in dairy cows [51]. IL-6 is a typical cytokine which helps to maintain homeostasis, but dysregulated excessive and persistent synthesis of IL-6 can have pathological effects on the acute systemic inflammatory response syndrome [52]. IL-lβ serves as a key mediator in the inflammatory response; it plays an essential role in host defense against pathogens but can also worsen damage during inflammatory responses [53]. TNF-α is an inflammatory cytokine produced by macrophages/monocytes during acute inflammation and is responsible for a variety of intracellular signaling events, leading to necrosis or apoptosis [54]. IL-18 regulates helper T-helper 1 (Th1) and T-helper 2 (Th2) responses, and is involved in inducing the production of Th2 cytokines by CD4+ T cells, natural killer cells, and Th1 cells [55]. Many previous studies have shown that LJE suppresses the inflammatory response by reducing the inflammatory factors, thereby supporting immune function [13]. In this study, the addition of LJE significantly reduced the production of inflammatory factors and increased IgG content in heat-stressed dairy cows, indicating that LJE can help to reduce the inflammatory response and improve immune function, with similar effects in the two LJE groups. Similarly, Zhao et al. [17] found that the addition of LJE significantly reduced serum IL-6 and IL-1β concentrations in perinatal cows. Additionally, it is worth noting that TNF-α, IL-6, IL-1β, and IL-18 are transcriptionally expressed through the activation of the NF-κB signaling pathway [56]. Substantial evidence supports CGA’s anti-inflammatory effects through the inhibition of the NF-κB signaling pathway [57,58], which might contribute to the anti-inflammatory effects of LJE.

The ruminal pH decreases during heat stress [59], which can inhibit the growth of pH-sensitive ruminal bacteria, such as cellulolytic bacteria, and lead to sub-acute ruminal acidosis [9]. In vitro tests conducted by Lee et al. [60] demonstrated that LJE improves rumen pH. Consistent with these findings, our study found that the addition of LJE increased rumen pH. The concentration of total VFA is considered as an indicator of energy balance and utilization in dairy cows, while the proportion of total VFA composition reflects the fermentation capacity of the rumen [61]. According to Bergman et al. [62], rumen fermentation accounts for approximately 70% to 80% of the energy absorbed by ruminants. Acetate, a major end product of rumen fermentation in dairy cows, is significantly reduced under heat stress conditions [63]. Our study demonstrated that the addition of LJE significantly increased both total VFA and acetate concentrations. Acetate serves as a primary carbon source for lipid synthesis in adipose and mammary tissues of ruminants [64], which may explain the increasing trend in milk fat content due to increased rumen acetate levels. Rumen-produced volatile fatty acids are utilized as a carbon backbone for MCP synthesis, providing 50% to 90% of bypass proteins that optimize protein utilization in dairy cows [65]. Increased MCP synthesis contributes to the mammary gland uptake of protein for milk protein synthesis [66], which might explain the higher milk protein in the LJE-10% CGA and LJE-20% CGA groups.

## 5. Conclusions

This study demonstrated that the addition of 35 g/(d·head) of LJE-10% CGA or 17.5 g/(d·head) of LJE-20% CGA both reduced RT, enhanced DMI, milk yield, milk composition, and improved rumen fermentation in high-yielding dairy cows experiencing heat stress. Through the analysis of the serum biochemical, antioxidant and immune indicators, we observed a reduction in CREA levels and increased antioxidant and immune function. Furthermore, while maintaining a consistent CGA content, the effects of the addition of both types of LJE are similar. These findings proved that the addition of LJE containing 4.1 g CGA/(d·head) effectively relieved heat stress and improved the lactation performance of heat-stress dairy cows by improving their antioxidant properties and immune functions, with CGA serving as the effective ingredient responsible for its anti-heat stress properties. This study offered a precise feeding method and dosage for the application of CGA in heat-stressed dairy cows.

## Figures and Tables

**Table 1:** Ingredients and chemical composition of the basal diet (% of as-fed DM).

Item	%
Ingredients	
Alfalfa hay	3.27
Oats	0.48
Corn silage	27.28
Syrup	2.19
Corn	20.83
Soybean meal	14.29
Flaked corn	12.27
Canola meal	3.39
Cottonseed	8.14
Extruded full-fat soybean	0.85
Yeast culture ^1^	1.44
NaHCO_3_	1.35
Limestone	1.11
NaCl	0.48
MgO	0.29
CaHPO_4_	0.29
Fat powder ^2^	1.00
Premix ^3^	1.05
Total	100
Nutrient levels ^4^	
Dry Matter	53.58
Crude protein	19.25
Ether extract	4.25
Ash	5.83
Neutral detergent fiber	32.24
Acid detergent fiber	18.40
Ca	0.64
P	0.45
NE_L_ ^5^ (Mcal/kg)	1.62

^1^ Diamond V XP yeast culture supplement (Diamond V, Cedar Rapids, IA). ^2^ Fat powder, a saturated free fatty acid supplement (Berg + Schmidt GmbH & Co. KG, Hamburg, Germany). ^3^ Premix provided 200,000 IU/kg vitamin A; 320,000 mg/kg vitamin B; 40,000 IU/kg vitamin D; 5000 IU/kg vitamin E; 100 mg/kg d-biotin; 95,200 mg/kg Mg; 1000 mg/kg Zn; and 50 mg/kg Co. ^4^ Analyzed value. ^5^ Calculated value (NE_L_ = 0.5501 × DE (Mcal/kg dry matter) − 0.3958, DE: Digestible Energy) [21].

**Table 2:** Effects of *Lonicera japonica* extract with different contents of chlorogenic acid on RT and RR in heat-stressed dairy cows.

Item	Treatment ^1^			SEM ^2^	*p* Value		
	Control	LJE-10% CGA	LJE-20% CGA		Trt	Week	Trt × Week
RT, °C	39.14 ^a^	38.97 ^b^	38.98 ^b^	0.04	0.02	<0.01	<0.01
RR, breaths/min	85.31	85.49	85.40	0.95	0.99	0.88	0.65

^a,b^ Mean values within treatment with different superscripts are significantly different (*p* < 0.05). ^1^ Treatment: CON (control), no LJE; LJE-10% CGA group, addition of 35 g/(d·head) LJE-10% CGA; LJE-20% CGA group, addition of 17.5 g/(d·head) LJE-20% CGA; n = 15 per group. ^2^ SEM, standard error of the mean. RT: rectal temperature; RR: respiratory rate.

**Table 3:** Effects of *Lonicera japonica* extract with different contents of chlorogenic acid on DMI, milk yield, and milk composition in heat-stressed dairy cows.

Item	Treatment ^1^			SEM ^2^	*p* Value		
	Control	LJE-10% CGA	LJE-20% CGA		Trt	Week	Trt × Week
DMI, kg/d	24.70 ^b^	25.89 ^a^	26.09 ^a^	0.38	0.03	0.91	0.99
Milk yield, kg/d	30.03 ^b^	31.805 ^a^	31.30 ^a^	0.50	<0.05	0.31	<0.01
Fat, %	3.46	3.56	3.56	0.04	0.09	0.34	0.98
Protein, %	3.23 ^b^	3.37 ^a^	3.43 ^a^	0.06	0.04	<0.01	<0.01
Lactose, %	4.85 ^b^	5.17 ^a^	5.21 ^a^	0.09	0.02	<0.01	0.04
SNF, %	9.05	9.19	9.33	0.13	0.28	<0.01	<0.01
TS, %	12.55	12.82	12.38	0.30	0.58	<0.01	<0.01
Feed efficiency	1.21	1.22	1.20	0.03	0.88	<0.01	0.21

^a,b^ Mean values within treatment with different superscripts are significantly different (*p* < 0.05). ^1^ Treatment: CON (control), no LJE; LJE-10% CGA group, addition of 35 g/(d·head) LJE-10% CGA; LJE-20% CGA group, addition of 17.5 g/(d·head) LJE-20% CGA; n = 15 per group. ^2^ SEM, standard error of the mean. DMI: dry matter intake; SNF: solids-not-fat; TS: lactose, total solids.

**Table 4:** Effects of *Lonicera japonica* extract with different contents of chlorogenic acid on serum biochemical index in heat-stressed dairy cows.

Item	Treatment ^1^			SEM ^2^	*p* Value
	Control	LJE-10% CGA	LJE-20% CGA		
Creatinine, μmol/L	76.69 ^a^	61.97 ^b^	62.25 ^b^	3.97	0.02
Uric acid, μmol/L	30.18	33.70	32.95	2.49	0.58
Urea nitrogen, mmol/L	3.65	4.03	4.35	0.26	0.17
Total protein, g/L	41.40	45.51	44.56	2.47	0.48

^a,b^ Mean values within treatment with different superscripts are significantly different (*p* < 0.05). ^1^ Treatment: CON (control), no LJE; LJE-10% CGA group, addition of 35 g/(d·head) LJE-10% CGA; LJE-20% CGA group, addition of 17.5 g/(d·head) LJE-20% CGA; n = 15 per group. ^2^ SEM, standard error of the mean.

**Table 5:** Effects of *Lonicera japonica* extract with different contents of chlorogenic acid on serum antioxidant indices in heat-stressed dairy cows.

Item	Treatment ^1^			SEM ^2^	*p* Value
	Control	LJE-10% CGA	LJE-20% CGA		
Glutathione peroxidase, U/mL	92.29 ^b^	100.01 ^a^	101.27 ^a^	2.15	0.01
Total antioxidant capacity, U/mL	4.16 ^b^	5.68 ^a^	5.95 ^a^	0.53	0.04
Superoxide dismutase, U/mL	100.38 ^b^	104.91 ^a^	106.36 ^a^	1.60	0.03
Malondialdehyde, nmol/mL	4.35 ^a^	3.14 ^b^	3.05 ^b^	0.25	0.01

^a,b^ Mean values within treatment with different superscripts are significantly different (*p* < 0.05). ^1^ Treatment: CON (control), no LJE; LJE-10% CGA group, addition of 35 g/(d·head) LJE-10% CGA; LJE-20% CGA group, addition of 17.5 g/(d·head) LJE-20% CGA; n = 15 per group. ^2^ SEM, standard error of the mean.

**Table 6:** Effects of *Lonicera japonica* extract with different contents of chlorogenic acid on serum immune indices in heat-stressed dairy cows.

Item	Treatment ^1^			SEM ^2^	*p* Value
	Control	LJE-10% CGA	LJE-20% CGA		
IgG, mg/mL	110.63 ^b^	117.15 ^a^	116.77 ^a^	0.54	0.01
IgA, mg/mL	0.79	0.81	0.80	0.05	0.96
IgM, mg/mL	3.74	3.78	3.80	0.26	0.99
IL-6, pg/mL	436.68 ^a^	350.26 ^b^	360.82 ^b^	14.28	0.01
TNF-α, pg/mL	286.14 ^a^	240.97 ^b^	235.94 ^b^	12.58	0.01
IL-18, pg/mL	43.91 ^a^	40.17 ^b^	39.89 ^b^	1.04	0.02
IL-1β, pg/mL	55.71 ^a^	46.57 ^b^	44.75 ^b^	3.05	0.03

^a,b^ Mean values within treatment with different superscripts are significantly different (*p* < 0.05). ^1^ Treatment: CON (control), no LJE addition; LJE-10% CGA group, addition of 35 g/(d·head) LJE-10% CGA; LJE-20% CGA group, addition of 17.5 g/(d·head) LJE-20% CGA; n = 15 per group. ^2^ SEM, standard error of the mean. IgG: immunoglobulin G; IgA: immunoglobulin A; IgM: immunoglobulin M; IL-6: interleukin-6; TNF-α: tumor necrosis factor-α; IL-18: interleukin-18; IL-1β: interleukin-1β.

**Table 7:** Effects of *Lonicera japonica* extract with different contents of chlorogenic acid on rumen fermentation in heat-stressed dairy cows.

Item	Treatment ^1^			SEM ^2^	*p* Value
	Control	LJE-10% CGA	LJE-20% CGA		
pH	5.97 ^b^	6.39 ^a^	6.39 ^a^	0.07	0.01
MCP, mg/mL	1.15 ^b^	1.32 ^a^	1.31 ^a^	0.43	0.03
Total VFA, mmol/L	83.97 ^b^	97.90 ^a^	94.33 ^a^	3.18	0.02
Acetate, mmol/L	47.29 ^b^	53.53 ^a^	54.71 ^a^	2.06	< 0.05
Propionate, mmol/L	24.44	28.10	26.30	1.26	0.15
Isobutyrate, mmol/L	0.72	0.79	0.76	0.05	0.60
Butyrate, mmol/L	9.49	10.53	9.62	0.75	0.57
Isovalerate, mmol/L	1.18	1.26	1.25	0.11	0.84
Valerate, mmol/L	1.52	1.68	1.53	0.15	0.69
Acetate/Propionate	1.99	1.92	2.10	0.14	0.67

^a,b^ Mean values within treatment with different superscripts are significantly different (*p* < 0.05). ^1^ Treatment: CON (control), no LJE addition; LJE-10% CGA group, addition of 35 g/(d·head) LJE-10% CGA; LJE-20% CGA group, addition of 17.5 g/(d·head) LJE-20% CGA; n = 6 per group. ^2^ SEM, standard error of the mean. MCP, microbial protein.

## Data Availability

Data are contained within the article.
